# Formal and informal help-seeking intentions/behaviors among students and workers during the COVID-19 pandemic: a scoping review

**DOI:** 10.1265/ehpm.23-00095

**Published:** 2023-09-22

**Authors:** Takashi Yamauchi, Takashi Shimazaki, Hiroyuki Yanagisawa, Machi Suka

**Affiliations:** 1 Department of Public Health and Environmental Medicine, The Jikei University School of Medicine, 3-25-8 Nishi-shimbashi, Minato-ku, Tokyo 105-8461, Japan; 2 The Jikei University School of Medicine, 3-25-8 Nishi-shimbashi, Minato-ku, Tokyo 105-8461, Japan

**Keywords:** Help-seeking intention, Help-seeking behavior, Student, Healthcare worker, COVID-19, Scoping review

## Abstract

**Background:**

Students and workers have been subjected to increased levels of psychological distress due to the quarantine policy and containment measures during the COVID-19 pandemic. This scoping review aimed to present an overview of published evidence regarding formal and informal help-seeking intentions/behaviors for non-mental health-related issues as well as mental health-related issues among students and workers during the pandemic.

**Methods:**

In June 2022, we searched MEDLINE, APA PsycNet, and CINAHL for articles reporting the state of help-seeking intentions/behaviors among students and workers during the pandemic. Peer-reviewed original articles published in English were selected.

**Results:**

In total, 150 articles were identified, and 12 articles were selected for final analysis after removing articles that met the exclusion criteria. Three studies targeted university students, and nine targeted healthcare workers. Study settings were restricted to Western countries and China. Of the 12 studies, 11 were observational and predominantly cross-sectional studies. Two longitudinal studies using student samples suggested that university students became more reluctant to seek help from both formal and informal sources during the COVID-19 pandemic compared to before, despite the increased need for support during the pandemic. Among healthcare workers, the proportions of those who sought help from formal sources in person were low (7–26%), even among those with mental health issues, despite the increase in the need for mental health services. One randomized controlled study reported that a brief video-based intervention increased treatment-seeking intentions among healthcare workers in the intervention group compared with the non-intervention group.

**Conclusions:**

The present review revealed that, although most studies included in the final analysis were cross-sectional, intentions/behaviors to seek help from both formal and informal sources decreased among university students, even those with mental health issues. Among healthcare workers, while the frequency of help-seeking from formal sources in person was low, a brief online intervention was suggested to be useful for promoting help-seeking from formal sources. During public health crises such as the COVID-19 pandemic, system and infrastructure development of online help-seeking services could potentially promote formal and informal help-seeking intentions/behaviors for diverse issues, including non-mental health-related issues, among university students and healthcare workers/providers.

**Supplementary information:**

The online version contains supplementary material available at https://doi.org/10.1265/ehpm.23-00095.

## Background

The promotion of help-seeking intentions/behaviors, which are generally defined as intentions/behaviors aimed at obtaining support and assistance from others to improve a situation or problem, to prevent severe psychological distress and suicidal behavior has been a major public and occupational health issue worldwide in recent years, especially during the peri-pandemic period of the novel coronavirus disease 2019 (COVID-19).

Among young people such as students, the proportion of individuals with severe psychological distress is high, and this trend has become more pronounced in recent years [1]. With respect to suicidal behavior, in high/upper-middle-income countries such as European and north American countries, the total number of suicide deaths has remained largely unchanged or declined in the early stage of the COVID-19 pandemic compared with the pre-pandemic period [2]. However, among students, suicide mortality rates have been on the rise in Japan, and these increases remained stable during the COVID-19 pandemic [3, 4]. Similarly, self-harm mortality rates increased by 1.4-fold among students aged 10–14 years in southern China during the pandemic period as compared with the pre-pandemic period [5, 6]. During the height of the COVID-19 pandemic, the quarantine policy and community containment measures necessitated that students study from home, and consequently, they experienced an increased level of psychological distress [7, 8] and lower engagement with learning [9]. In most countries, opportunities for students to communicate with each other decreased due to the closing of schools/universities and the implementation of remote teaching and e-learning systems. Meanwhile, young people tend to avoid seeking professional help for mental health issues from “formal” sources and use more “informal” sources of help, such as family members and friends, compared with older people [10, 11].

Among workers, studies on help-seeking behaviors related to health issues have primarily focused on specific industries or occupations [12–14], such as military personnel, medical professionals, police workers, ambulance personnel, and firefighters. In our previous study, we examined associations between help-seeking behavior and psychological well-being among workers in various occupations using a nationally representative sample of Japanese workers, and suggested that help-seeking behavior has a preventive buffer effect against psychological distress regardless of age [15]. The widespread application of telework/telecommuting changed the way of working as well as the frequency and nature of direct communication among workers, especially clerical/sales workers, during the COVID-19 pandemic [16–18].

In response to the increasing importance of help-seeking during the COVID-19 pandemic, Yonemoto et al. recently conducted a systematic literature review to examine the state of help-seeking behaviors during the COVID-19 pandemic and revealed delays and decreases in help-seeking behaviors from mental health professionals among patients [19]; however, the focus of their review was on formal help-seeking behaviors for only mental health-related problems, and studies regarding formal and informal help-seeking intentions/behaviors for non-mental health-related issues, such as family, job, and academic-related issues, were excluded. Thus, we conducted a comprehensive literature search with the aim of reviewing and summarizing published evidence regarding the state of formal and informal help-seeking intentions/behaviors for non-mental health-related issues as well as mental health-related issues among students and workers during the COVID-19 pandemic, since both students and workers have been subjected to increased levels of psychological distress due to the quarantine policy and containment measures such as e-learning and telework systems during the pandemic. Understanding what is currently known on this topic may contribute to promoting factors associated with formal and informal help-seeking during the ongoing and future public health crises and provide useful implications for future studies on this topic.

## Methods

### Literature search strategy

Given the extensive and heterogeneous nature of the literature on help-seeking intentions/behaviors among students and workers during the COVID-19 pandemic, we conducted a scoping review that does not require protocol registration in accordance with the Preferred Reporting Items for Systematic reviews and Meta-Analyses extension for Scoping Reviews (PRISMA-ScR) statement and checklist [20]. Ethical approval was not sought, as the present study used only information available in published articles.

On June 25, 2022, we searched MEDLINE, APA PsycNet (includes PsycINFO and PsycARTICLES), and CINAHL for articles published in English using the following search terms: “workers,” “employees,” “personnel,” “students,” “help-seeking,” “seek help,” “COVID-19,” and “coronavirus.” Our database search strategy is presented in [Additional file 1]. To further identify relevant publications, we also examined the reference lists of all articles selected for full-text assessment.

### Inclusion and exclusion criteria

The first two authors independently examined the abstracts of all articles identified in the search. Inclusion criteria were peer-reviewed original articles, articles published in English, and articles reporting the state of help-seeking intentions/behaviors among students and/or workers during the COVID-19 pandemic. Subsequently, full texts were screened to exclude articles that met the following criteria: (1) did not concern help-seeking intentions/behaviors during the pandemic as a primary or secondary outcome measure, (2) did not contain a detailed description of participants’ demographics, study methods, and/or primary data on help-seeking, (3) analyzed data obtained from only individuals with physical/mental health issues, including suicidal ideation/attempts, and (4) analyzed data that had been obtained before the onset of the COVID-19 pandemic. In addition, if the same original dataset was used in multiple studies, only articles that used the most comprehensive sample were included in the final analysis. There was no restriction on articles based on the study design.

### Data charting

Three investigators developed a data charting form, which was used to extract the following information from all eligible articles: data source (i.e., name of authors and publication year), study setting, objectives, participants, study design and methods, outcome/measurement/definition (i.e., help-seeking intentions/behaviors), and main study findings. As previous studies suggested [19], there is no gold standard for measuring help-seeking behavior, and terms regarding help-seeking vary between studies. Thus, in Table 1, we included the measurement and definition of help-seeking intentions/behaviors used in each study.

**Table 1:** Data from selected articles

	**Source**	**Country**	**Study aims**	**Participants**	**Study design / Methods**	**Outcome / Measurement / ** **Definition**	**Main findings**
***Student sample studies***							
1	Zhan, 2022	China	To explore whether there was any change in college students’ experience of family harmony, including help-seeking from family members, before and after the COVID-19 outbreak	215 undergraduates from a university in Tianjin (119 females, 55%)	Longitudinal study; Questionnaire surveys in Dec 2019 and Mar 2020	Help-seeking intentions/behavior for diverse issues from family members; Defined according to the “Help-seeking” dimension of the College Students’ Experience of Family Harmony Questionnaire (CSEFHQ) (5 items)	Help-seeking from family members significantly decreased after onset of the pandemic

2	Conceição, 2021	Portugal	To evaluate the effect of the pandemic, including lockdown measures, on mental health care seeking among university students	366 students from a university in Porto (261 females, 71%)	Longitudinal study; Online questionnaire surveys at three time points (Oct 2019, Jun 2020, and Mar 2021)	Presence/absence of access to mental health care/treatment-seeking; No statement regarding the definition of help-seeking	Despite the increase in the severity of depressive/anxiety symptomatology, care-seeking behaviors did not change (20%, 23%, and 21% of participants in Oct 2019, Jun 2020, and Mar 2021, respectively); More than half of participants with mild/severe symptomatology did not seek treatment during the pandemic, and this proportion was significantly higher than that before the pandemic

3	Liang, 2020	China	To investigate the relationship between mental health status and psychological help-seeking behavior among college students	4,164 students from universities in Guangdong Province (2,000 females, 48%); Participants were assigned to the “counseling group” and “non-counseling group” according to whether they had sought psychological help due to the COVID-19 outbreak	Cross-sectional; An online questionnaire survey in Feb 2020	Psychological help-seeking in response to the COVID-19-related situation; Defined based on a single-item question “Have you ever sought psychological assistance in response to the COVID-19 epidemic situation?”	11% of respondents had sought psychological help from professionals (e.g., counselors) in the past; Participants who had poor mental health status and past experiences of receiving psychological help service were more likely to seek psychological help in response to the crisis

***Worker sample studies***							
1	Bismark, 2022	Australia	To examine the state of (a) thoughts of suicide/self-harm among healthcare workers and (b) help-seeking behavior among those with suicidal thoughts during the pandemic	7,795 healthcare workers (6,300 females, 81%) of all professions, who were self-identified as frontline healthcare workers in primary or secondary care	Cross-sectional; A nationwide online survey, the Australian COVID-19 Frontline Healthcare Workers Study, between Aug and Oct 2020	Informal help-seeking (i.e., use of psychological health apps) and help-seeking behavior from professionals (i.e., a doctor/psychologist, employee support program, or other professional support program outside of work) for mental health issues; Defined based on a single-item question “Since the COVID-19 pandemic started, have you sought help from any of the following sources for stress, anxiety, depression or another mental health issue?”	11% of participants reported suicidal thoughts; 39% and 16% of participants with and without thoughts of suicide/self-harm, respectively, reported that they had sought help from a doctor/psychologist

2	Amsalem, 2022	US	To evaluate the efficacy of a brief, social contact-based video intervention in increasing treatment-seeking intentions among healthcare workers during the pandemic	350 US-resident healthcare workers (260 females, 74%), including nurses (68%) and physicians (15%)	Randomized controlled trial; Participants were randomized to a 3-min video-intervention group, a repeated video-intervention group, and a non-intervention control group. Treatment-seeking intentions were assessed pre/post-intervention and at 14- and 30-day follow-ups	Treatment-seeking intentions; Defined based on “Openness to help-seeking” of the Attitudes towards Seeking Professional Psychological Help Scale (ATSPPH-SF) (3 items)	80% of participants reported probable psychopathology (i.e., anxiety, depression, and/or PTSD); A brief video-based intervention increased treatment-seeking intentions in the two intervention groups, particularly among participants in the repeated video intervention group

3	Ménard, 2022	Canada	To assess the use of formal/informal mental health supports and reasons for non-use of these resources among hospital employees during the pandemic	650 healthcare workers at an acute care hospital in Ontario (539 females, 83%), including nurses (51%), allied professionals (17%), and clerical workers (16%)	Cross-sectional; An online survey between Nov and Dec 2020	Use of formal/informal mental health supports; Defined as a list of support resources, including COVID-specific teletherapy, employee assistance program, public helplines, in-person counselling/therapy, online self-help, formal support groups, informal peer support, and other forms	Use of any formal mental health supports was low (20%), with the most frequently-reported reason for not seeking supports being “problems not severe enough to require this service”; 77% of participants reported seeking informal peer support, of whom 72% found the support to be effective

4	Salgado de Snyder, 2021	US	To analyze occupational stress, mental health, and help-seeking behaviors among healthcare workers/providers serving socially vulnerable groups such as immigrants, refugees, and people living in poverty during the pandemic	407 clinic-based healthcare workers/providers affiliated with two national organizations in the United States serving socially vulnerable populations (females 87%), including community health workers (49%) and health providers (nurses/physicians 19%)	Cross-sectional; A self-administered online survey between Jul and Sep 2020	Help-seeking behavior from mental health professionals (in-person and/or online) and self-care behaviors; Defined based on a dichotomous single-item question about if they sought support from a mental health professional in-person and/or virtually during the pandemic	While 7% of participants sought mental health support from a professional in person, 23% did so virtually; 52% reported seeking emotional support from family/friends in an attempt to find comfort and psychological well-being

5	Smallwood, 2021	Australia	To investigate coping strategies and help-seeking behaviors, as well as their association with mental health symptoms, among frontline healthcare workers during the pandemic	7,846 frontline healthcare workers in primary/secondary care settings (nursing 39%, medical 31%); participants were predominantly females (81%), and 52% were younger than 40 years	Cross-sectional; A nationwide online survey, the Australian COVID-19 Frontline Healthcare Workers Study, which was conducted between Aug and Oct 2020	Help-seeking behavior for mental health issues from professionals; Defined based on a multiple-choice question on the presence/absence of help-seeking for mental health issues from professional sources (i.e., a doctor/psychologist, employee support program at workplace, professional support program outside of work, or other) “Since the COVID-19 pandemic started, have you sought help from any of the following sources for stress, anxiety, depression or another mental health issue?”	74% of participants did not seek any psychological help from professionals or accessing professional support programs; Participants with a prior mental health diagnosis were more likely to utilize psychological support services

6	She, 2021	China	To examine the state of mental health help-seeking behavior and associated factors among public health workers who are devoted to COVID-19 control and prevention work during the pandemic	3,417 public health workers (2,262 females, 66%) who reported probable mental health concerns among 9,475 potential participants in five provinces across China, predominantly engaged in primary care or disease control/health education	Cross-sectional; An online survey between Feb and Mar 2020	Mental health help-seeking from professionals; Defined by a single-item question about whether the participants had managed to seek help from mental health professionals during the COVID-19 outbreak phase (yes/no)	Whereas 41% of participants reported probable depression, only 13% reported professional mental health help-seeking; Participants who received psychological training, perceived a higher level of support from the society, and had depression/anxiety were more likely to seek help; The belief that mental health issues were not the priority (64%) and lack of time (56%) were among the most frequent reasons for not seeking help

7	Weibelzahl, 2021	Germany	To investigate (1) the prevalence of work-related stressors, (2) psychological effects of these stressors on clinical symptoms, and (3) help-seeking intentions among healthcare professionals during the pandemic	300 healthcare professionals in Germany (240 females, 81%), of whom nearly one third were engaged in inpatient nursing care	Cross-sectional; An online survey between May and Jul 2020	Psychological help-seeking intentions (e.g., psychotherapy) for psychological strain; Defined based on a self-formulated question about whether participants would consider using different types of mental support	While 70% of participants described themselves as experiencing symptoms of depression/anxiety, only 40% of them reported they would consider seeking psychological support; Participants with stronger depression/anxiety symptoms were more likely to seek psychological support

8	Cai, 2020	China	To compare the impact of the COVID-19 outbreak on mental health issues, including help-seeking or treatment for mental health, between frontline and non-frontline medical workers during the pandemic	1,173 frontline medical workers (i.e., those dealing with COVID-19) and 1,173 age/sex-matched non-frontline medical workers working in hospitals in China; participants were predominantly females (70%)	Cross-sectional; An online questionnaire survey in Feb 2020	Mental health treatment-seeking from professionals; Defined based on two single-item questions “Have you ever sought help from psychiatrists/clinical psychologists since the outbreak of COVID-19 began?” and “Have you ever received any treatment for psychiatric/psychological problems since the outbreak began?”	While frontline medical workers had a higher rates of any mental health problems compared with their non-frontline counterparts (53% vs 34%), among those with such problems, no significantly higher rates of seeking help from professional (5% vs 5%) or receiving treatment (3% vs 2%) were observed

9	Lai, 2020	China	To explore the level and related factors of help-seeking behavior regarding returning to work in healthcare workers during the pandemic	861 healthcare workers (789 females, 92%) working in a hospital in Wuhan, China, predominantly as nurses (81%)	Cross-sectional; An online questionnaire survey (no information about the survey period)	Help-seeking from formal and informal resources (i.e., sources from outside the hospital) for four specified problems (i.e., a diagnosis of COVID-19, medical service (e.g., admission process), work schedule, and infection control); Defined as any action or activity carried out by a person who perceives herself/himself in need of personal, psychological, or affective assistance, or health or social service; A dichotomous single-item question about whether the participants had sought help for the specified problems and how likely they would seek help for a specified problem	17% of participants sought help, and problems they sought help the most for were COVID-19 diagnosis-related problems; Percentages of help-seeking behavior ranged from 2 to 5% for informal sources and 25 to 37% for formal sources; Regardless of the type of problem, help-seeking from formal sources was more frequent than that from informal sources

## Results

**Fig. 1:**
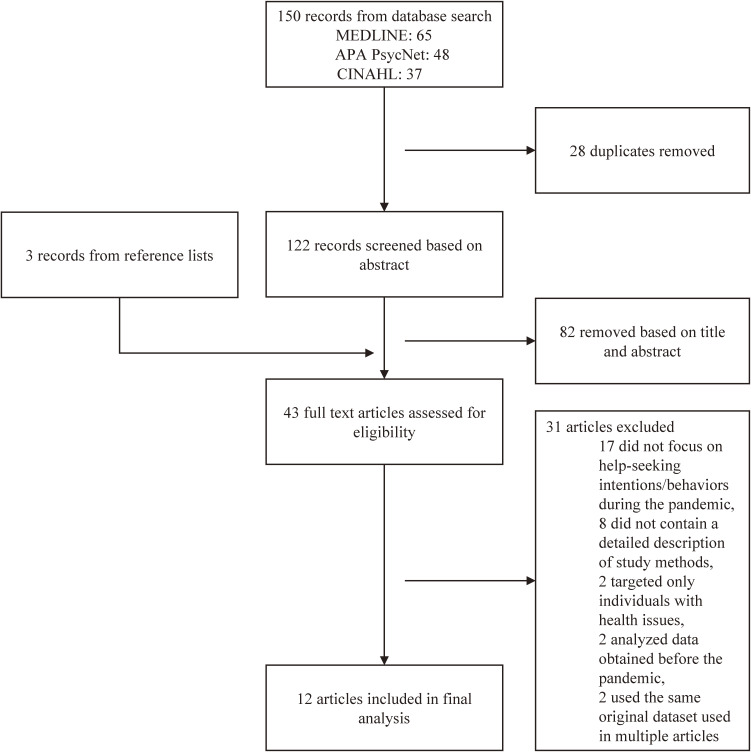
Flowchart of article selection for final analysis

Our literature search strategy returned 150 publication records (Fig. 1). After removing duplicates and assessing articles based on the title and abstract, 43 were selected for full-text review. Of the 43 articles, 31 that met the selection criteria were excluded. The remaining 12 original articles were selected for the final analysis. Table 1 provides a summary of these articles by study sample (i.e., student sample and worker sample studies).

### Participant characteristics and study settings

Three (25%) studies focused on university students [9, 21, 22], and nine (75%) focused on workers in medical/health/welfare industries, including hospital-based healthcare workers and community/public health workers, who were predominantly female (66% to 87% of the total sample) [23–31]. There were no studies that focused on other types of students (e.g., high school students) or workers (e.g., workers in manufacturing/construction industries). Healthcare worker samples in the selected studies were primarily nursing staff.

Two studies were conducted in Australia [23, 24], one in Canada [26], five in China [21, 22, 28, 30, 31], one each in Germany [29] and Portugal [9], and two in the United States [25, 27]. Study sample sizes ranged from 215 to 7,846, with a median of 756 (interquartile range, 3,623).

### Study design

Of the 12 studies, 11 were observational studies, predominantly cross-sectional in design, and only two were longitudinal studies with relatively small samples of university students [9, 21]. Both longitudinal studies used a pre-pandemic time point as the baseline to examine changes in help-seeking intentions/behaviors among university students during the pandemic. Most observational studies used an online questionnaire survey for data collection; there was no secondary data analysis study.

We also identified one randomized controlled trial evaluating the effect of social contact-based video intervention on treatment-seeking intentions with a 30-day follow-up period among US-resident healthcare workers [25].

### Outcome/measurement

Eight studies focused on help/treatment-seeking behaviors [9, 22–24, 26–28, 30], two focused on help/treatment-seeking intentions [25, 29], and two focused on both [21, 31]. All studies used a self-rating questionnaire method to assess the presence/absence of help-seeking intentions/behaviors. While nine studies investigated help-seeking from mental health professionals in-person or online during the pandemic and used an originally developed single-item question regarding help-seeking intentions/behaviors, three studies examined help-seeking for non-mental health-related issues such as COVID-19-specific problems [21, 22, 31]. Three studies also examined help-seeking from informal sources, such as family members and peers [23, 26, 31].

### Key findings

#### Student sample studies

A longitudinal study in China using a sample of university students revealed that help-seeking from family members significantly decreased between pre- and peri-pandemic periods [21]. Another longitudinal study in Portugal showed that, despite the increase in the severity of depressive/anxiety symptomatology, help-seeking behaviors among university students did not change between pre- and peri-pandemic periods (20%, 23%, and 21% of participants in Oct 2019, Jun 2020, and Mar 2021, respectively) [9]. Moreover, more than half of participants with mild/severe symptomatology did not seek treatment during the pandemic; this proportion was significantly higher compared with that before the pandemic.

#### Worker sample studies

In a randomized controlled study, 80% of 350 US-resident healthcare workers, predominantly nurses, reported probable psychopathology (e.g., anxiety, depression, PTSD) during the pandemic [25]. That study also showed that a brief video-based intervention, which used a 3-min video in which a female nurse described difficulty coping with COVID-19-related stress, her depression/anxiety, barriers to treatment, and how psychotherapy helped her, increased treatment-seeking intentions in the repeat-video group (i.e., a video-based intervention at day 1 coupled with a booster intervention at day 14) compared with the non-intervention group.

In cross-sectional studies using healthcare worker samples [23, 24, 26–31], the use of formal mental health support in person was low (e.g., 7% of total participants in a US study [27]). A study in China reported that 41% of participants reported probable depression, whereas only 13% reported professional mental health help-seeking [28]. Another study in China reported that, even in a subsample of participants who reported probable mental health concerns, less than 10% reported mental health help-seeking from professionals [30]. Regarding suicidal ideation, one study using a nationwide cross-sectional design in Australia investigated suicidal ideation among frontline healthcare workers during the pandemic, and reported that, while 11% of participants reported suicidal ideation, only 39% of those with suicidal ideation had sought help from a doctor/psychologist [23].

## Discussion

This scoping review aimed to present an overview of published evidence regarding formal and informal help-seeking intentions/behaviors for non-mental health-related issues as well as mental health issues among students and workers during an ongoing public health crisis (i.e., the COVID-19 pandemic).

### Summary of key findings and interpretations

#### Student sample studies

Of the two longitudinal studies using student samples, one reported a significant decrease in help-seeking from family members among university students during the pandemic compared to before [21], whereas the other found no change in treatment-seeking behaviors among university students from different schools/courses between pre- and peri-pandemic periods [9]. However, the latter study also showed that despite the increase in the severity of depressive/anxiety symptomatology during the pandemic, more than half of participants with mild/severe symptomatology did not seek care from mental health professionals; this proportion was significantly higher than that before the pandemic. These findings suggest that, while the need for adequate support and care increased during the pandemic, help-seeking behaviors among university students decreased, even among those with mental health issues. Although the two aforementioned studies were conducted in restricted regions (i.e., China and Portugal) with small sample sizes, they suggest that changes in social situations due to pandemic measures, including strict social distancing and “stay-home” (i.e., lockdown) orders, might have made university students feel more hesitant to seek help from formal and informal sources than they did before the pandemic. As for help-seeking from informal sources, following the widespread adoption of remote teaching and e-learning due to the pandemic, university students would not choose to talk to or ask for advice from their families, thinking that they would not be of any help when it comes to solving academic-related problems and difficulties [21]. In sum, these findings imply that university students became more reluctant to seek help from both formal and informal sources after the onset of the pandemic.

#### Worker sample studies

A randomized controlled study conducted in the United States showed that a brief video-based intervention, which used a 3-min video of a nurse describing her COVID-19-related stress and help-seeking from professionals, increased treatment-seeking intentions among healthcare workers in the intervention group compared with the non-intervention group [25]. In that study, participants with probable psychopathology, including PTSD, accounted for 80%, emphasizing the strong need for interventions to enhance help-seeking intentions/behaviors. Previous studies have indicated that healthcare workers tend to be reluctant to seek help or support [32–34]. The barriers against help-seeking for mental health problems among healthcare workers include their stigmatized attitude toward psychological/psychiatric treatment, a perception of treatment-seeking as a weakness, and concerns about negative evaluations in the workplace. In addition, the ongoing COVID-19 pandemic has intensified stress, including stress due to fear of getting sick and financial issues, among employees working for medical institutions and communities. As the COVID-19 pandemic continues, brief online interventions using videos or apps to increase treatment-seeking intentions/behaviors could potentially promote the use of mental health support among healthcare workers/providers.

In cross-sectional studies using healthcare worker samples [23, 24, 26–31], access to mental health professionals and the use of in-person psychological/psychiatric services were low (i.e., 7–26% of all participants). Even in a subsample of participants who reported probable mental health concerns or suicidal thoughts, most participants did not report mental health help-seeking from health professionals [23]. Some studies investigated the state of help-seeking intentions/behaviors among healthcare workers by occupation (e.g., physicians or nurses) and showed that, regardless of occupation, seeking formal help for mental health issues was uncommon among healthcare workers, and that common predictors for help-seeking from a doctor/psychologist were female sex and having a prior mental health diagnosis [35, 36].

All nine studies using worker samples focused on healthcare workers in medical institutions or communities, who have been reported to be reluctant to seek help from health professionals [33]. During the pandemic, the widespread application of telework/telecommuting changed the way of working among workers, especially white-collar workers, worldwide [18, 37, 38]. For those workers, the use of telework systems during the pandemic may have lowered the threshold for seeking help from their companies and supervisors/colleagues [39, 40], as these systems allowed them to continue working remotely and communicate with their supervisors/colleagues online. While many companies introduced telework systems during the pandemic, most workers in medical/health/welfare industries have been forced to keep doing their job in person in their workplace, even under the ongoing pandemic, because of the nature of their occupational duties. As these factors are likely to influence help-seeking intentions/behaviors, changes in help-seeking intentions/behaviors during the pandemic may differ by occupation and industry.

Healthcare workers who participated in the studies included in the present review were predominantly female. Previous studies have shown that young individuals tend to avoid seeking professional help for mental health issues and use more informal sources of help, such as family members, parents, and friends, compared with older persons [10, 11], and that help-seeking behaviors tend to be more frequent among females than males [41]. Future studies should focus on help-seeking intentions/behaviors for non-mental health-related issues as well as mental health issues among male healthcare workers, especially older males, during public health crises.

### Comparison of findings from student and worker sample studies

The present scoping review suggests that, for both university students and healthcare workers, seeking formal help for mental health issues was uncommon; regardless of the type of problems, help-seeking from informal sources was more frequent than help-seeking from formal sources. In addition, those who had past experiences of receiving professional psychological services were more likely to seek psychological help in response to COVID-19-related crises. However, it was difficult to compare the findings obtained from student sample studies with those obtained from worker sample studies because (1) measurements of formal and informal help-seeking intentions/behaviors differed from study to study, with most studies evaluating help-seeking intentions/behaviors using an originally developed single-item question; and (2) there were only three student sample studies, of which two were longitudinal studies with small sample sizes, whereas all worker sample studies were cross-sectional in design except for one intervention study [25].

### Strengths and limitations

To our knowledge, this is the first exploratory scoping review based on the literature focusing on the state of formal and informal help-seeking intentions/behaviors for problems including non-mental health-related issues among students and workers during the COVID-19 pandemic. While a previous literature review regarding the state of help-seeking behaviors during the COVID-19 pandemic focused on help-seeking behaviors for only mental health-related issues [19], the present scoping review comprehensively addressed formal and informal help-seeking intentions/behaviors for mental health-related issues as well as non-mental health-related issues, including family and school-related issues. Four major publication databases were searched to identify articles that specifically targeted student and worker samples.

This study also has some limitations worth noting. First, we did not consider publication biases or assess the quality of each study included in the final analysis. Although quality assessment of individual studies is not a required element of scoping reviews unlike systematic reviews and meta-analyses, the quality and reliability of the body of evidence was generally low for the outcomes assessed in the selected studies; most studies were cross-sectional in design, and help-seeking intentions/behaviors were evaluated with an originally developed single-item question using an online questionnaire survey method. Sample sizes of the selected studies were also relatively small, especially the longitudinal studies using university student samples [9, 21].

Second, while we searched four major databases including APA PsycNet to identify articles, other databases, such as EMBASE, were not used. However, since help-seeking intentions/behaviors are studied mostly in the field of psychology, we think that essential articles had been screened and included in the final analysis.

Third, study settings were restricted to China and Western countries. Help-seeking intentions/behaviors among students and workers during the pandemic likely vary across countries and even across geographical areas within a country, given differences in comprehensive social situations such as national healthcare and insurance systems, the severity of the impact of the pandemic on the society/community, and socio-economic and cultural factors. Indeed, previous studies have shown that negative and stigmatizing public/cultural attitudes toward people with mental health problems have formed a prominent barrier against help-seeking [42], and that the degrees of these stigmatized attitudes varied between countries/ethnicities [43]. Previous systematic reviews have also suggested that several socio-economic and cultural factors, such as low mental health literacy, influence help-seeking behaviors for psychological distress [44, 45].

Fourth, measurements of help-seeking intentions/behaviors differed from study to study, with most studies evaluating actual behaviors using an originally developed single-item question. Although we intended to collect a wide range of articles using general search phrases, it is possible that our search strategy might have missed some articles regarding help-seeking intentions/behaviors during the COVID-19 pandemic, as there is no gold standard for measuring help-seeking behaviors [19].

### Future perspective

First, regarding the sources of help-seeking, most studies included in the present review investigated only help-seeking from mental health professionals, such as psychiatrists and psychologists, in person or online during the pandemic. In contrast, among young people, the willingness to use informal sources for help-seeking or self-help strategies is a key component of mental health literacy [46, 47]. However, the present review revealed that only three studies have examined help-seeking from informal sources, such as family members and peers [21, 23, 26], and only three studies have investigated help-seeking for non-mental health-related issues such as COVID-19-specific problems [21, 22, 31]. A longitudinal study in China using a student sample reported that help-seeking from family members significantly decreased after the onset of the pandemic [21]. On the other hand, a cross-sectional study in Canada indicated that, although the proportion of those who used formal mental health support was low (i.e., approximately 20% of all participants), nearly 80% of healthcare workers sought informal peer support, and of these, 70% found such help-seeking to be effective [26]. Previous studies have shown that the sources of help-seeking for mental health problems vary across age groups; young individuals tend to avoid seeking professional assistance for mental health issues and use more informal sources [10, 11]. Thus, future studies should examine the state of help-seeking for non-mental health-related issues as well as mental health-related issues from informal sources in the general population under the social context that forces people to stay away from close contact and keep distance from intimate persons and social activities (e.g., the COVID-19 pandemic).

Second, a previous study in the US reported that, while 7% of healthcare workers sought professional mental health support in person, 23% did so virtually peri-pandemic [27]. Although this is most likely due to the influence of COVID-19 measures, healthcare workers/providers who tend to be reluctant to seek help or support [32–34] may find treatment-seeking behaviors via the Internet to be more favorable and comfortable than seeking treatment in person. The findings of the present scoping review suggest that, while the need for adequate support and care increased during the pandemic, help-seeking behaviors among university students and healthcare workers decreased, even among those with mental health issues. Furthermore, changes in social situations due to pandemic measures, especially strict social distancing orders, might have made people feel more hesitant to seek help from formal and informal sources in person than they did before the pandemic. Thus, during public health crises such as the COVID-19 pandemic, system development to increase the utility of Internet-based help-seeking, including social networking services and smartphone apps, could promote both formal and informal help-seeking among university students and healthcare workers.

## Conclusions

The present scoping review revealed that, despite the increased need for adequate support and care during the COVID-19 pandemic, help-seeking from both formal and informal sources decreased among university students, even those with mental health issues. Among healthcare workers, while the frequency of help-seeking from formal sources in person was low, a brief online intervention was suggested to be useful for promoting help-seeking from formal sources. During public health crises such as the COVID-19 pandemic, system and infrastructure development of online help-seeking services using, for instance, social networking services and smartphone apps, could potentially promote formal and informal help-seeking intentions/behaviors for diverse issues, including non-mental health-related issues, among university students and healthcare workers/providers.

## Abbreviations

COVID-19: coronavirus disease 2019
PRISMA-ScR: the Preferred Reporting Items for Systematic reviews and Meta-Analyses extension for Scoping Reviews

## Supplementary information

**Additional file 1:** Database search strategy.
