# Supplementary material for: Formal and informal help-seeking intentions/behaviors among students and workers during the COVID-19 pandemic: a scoping review
Source: Environ Health Prev Med. 2023 Sep 22;28:53. doi: 10.1265/ehpm.23-00095 (PMC10519802; doi:10.1265/ehpm.23-00095)
Supplement: Supplementary file 1 — Additional file 1: Database search strategy. [file ehpm-28-053-s001.docx]

**Additional file 1. Database search strategy**

MEDLINE

(workers OR employees OR personnel OR students) AND (help-seeking OR "seek help") AND (covid-19 OR coronavirus) AND "english"[Language]

APA PsycNet

(workers OR employees OR personnel OR students) AND (help seeking OR seek help) AND (covid-19 OR coronavirus)

CINAHL

(workers OR employees OR personnel OR students) AND (help-seeking OR "seek help") AND (covid-19 OR coronavirus)
